# Society for Cardiovascular Magnetic Resonance (SCMR) guidance for re-activation of cardiovascular magnetic resonance practice after peak phase of the COVID-19 pandemic

**DOI:** 10.1186/s12968-020-00654-8

**Published:** 2020-08-10

**Authors:** Bradley D. Allen, Timothy C. Wong, Chiara Bucciarelli-Ducci, Jennifer Bryant, Tiffany Chen, Erica Dall’Armellina, J. Paul Finn, Marianna Fontana, Marco Francone, Yuchi Han, Allison G. Hays, Ron Jacob, Chris Lawton, Warren J. Manning, Karen Ordovas, Purvi Parwani, Sven Plein, Andrew J. Powell, Subha V. Raman, Michael Salerno, James C. Carr

**Affiliations:** 1 Department of Radiology, Northwestern University Feinberg School of Medicine, Chicago, IL USA; 2 Department of Medicine (Cardiology), University of Pittsburgh School of Medicine, Pittsburgh, PA USA; 3 Bristol Heart Institute, Bristol NIHR Biomedical Research Centre, University Hospitals Bristol and University of Bristol, Bristol, UK; 4 National Heart Research Institute Singapore, National Heart Center Singapore, 5 Hospital Drive, Singapore, Singapore; 5 Cardiovascular Division, Perelman School of Medicine, University of Pennsylvania, Philadelphia, PA USA; 6 Leeds Institute of Cardiovascular and Metabolic Medicine, Department of Biomedical Imaging Sciences, University of Leeds, Leeds, UK; 7 Departments of Radiology and Medicine, UCLA, Los Angeles, California USA; 8 Division of Medicine, University College London, London, UK; 9 Department of Radiological, Oncological and Pathological Sciences, Sapienza University of Rome, Rome, Italy; 10 Departments of Medicine (Cardiovascular Division) and Radiology, Perelman School of Medicine, University of Pennsylvania, Philadelphia, PA USA; 11 Department of Medicine, Johns Hopkins University, Baltimore, MD USA; 12 The Heart and Vascular Institute, Lancaster General Health/PENN Medicine, Lancaster, PA USA; 13 Departments of Medicine (Cardiovascular Division) and Radiology, Beth Israel Deaconess Medical Center, Harvard Medical School, Boston, MA USA; 14 Departments of Radiology and Medicine, University of California, San Francisco, San Francisco, California USA; 15 Department of Medicine (Cardiology), Loma Linda University, Loma Linda, California USA; 16 Department of Cardiology, Boston Children’s Hospital, Boston, MA USA; 17 Department of Pediatrics, Harvard Medical School, Boston, MA USA; 18 Krannert Institute of Cardiology, Indiana University School of Medicine, Indianapolis, Indiana USA; 19 Departments of Medicine, Radiology, and Biomedical Engineering, University of Virginia, Charlottesville, Virginia USA

**Keywords:** COVID-19, Cardiovascular magnetic resonance, Workflow, Safety

## Abstract

During the peak phase of the COVID-19 pandemic, alterations of standard operating procedures were necessary for health systems to protect patients and healthcare workers and ensure access to vital hospital resources. As the peak phase passes, re-activation plans are required to safely manage increasing clinical volumes. In the context of cardiovascular magnetic resonance (CMR), re-activation objectives include continued performance of urgent CMR studies and resumption of CMR in patients with semi-urgent and elective indications in an environment that is safe for both patients and health care workers.

## Introduction

The global pandemic caused by severe acute respiratory syndrome coronavirus 2 (SARS-CoV-2) and the resultant clinical syndrome COVID-19 [1, 2] has now spread to 188 countries with over 11.2 million infections and over 531,000 deaths around the world as of July 5, 2020 [3]. Throughout the evolution of the pandemic, most regions have experienced a surge of cases which threatened to overwhelm vital health system resources including intensive care facilities, ventilators, and personal protective equipment (PPE) for frontline health care workers [4]. In order to aid virus containment efforts, in the United States, the Centers for Medicare and Medicaid Services (CMS) recommended that non-essential medical procedures be delayed during the peak-phase of the pandemic [5]. Similarly, both the European Society of Radiology (ESR) and European Society of Cardiac Radiology (ESCR) explicitly recommended to post-pone non-urgent procedures and restrict imaging services to those indications having an immediate impact on patient’s management, as the imaging procedure interrupts social distancing and potentially exposes to infection [6, 7]. Most health systems have responded by significantly limiting non-urgent and elective medical care including elective diagnostic imaging [8].

**Fig. 1:**
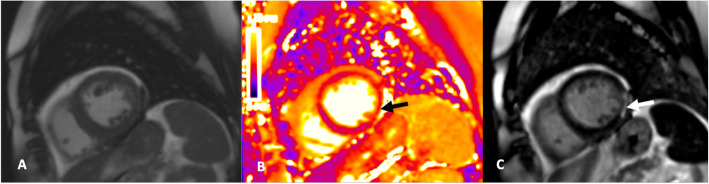
Example cardiovascular magnetic resonance (CMR) images of a patient with COVID-19 infection and acute myocarditis. Short axis cine of the basal infero-lateral wall (**a**), corresponding T2-map (**b**) demonstrating elevation of T2 indicating presence of edema (black arrow) and phase sensitive inversion recovery (PSIR) late gadolinium enhancement (LGE) (**c**) showing sub-epicardial enhancement (arrow)

The Society for Cardiovascular Magnetic Resonance (SCMR) has recently provided guidance on best practices for cardiovascular magnetic resonance (CMR) utilization during the COVID-19 pandemic [9]. As the first peak is passing in many locations around the world, some local governments and health care systems have been encouraged to slowly and safely transition to pre-COVID-19 health care delivery volumes [10]. Further, there is increasing recognition of CMR’s unique ability to assess COVID-19-related cardiovascular complications in a single non-invasive examination. This next phase requires further guidance on the safe and effective practice of CMR highlighted by continued timely performance of urgent CMR studies, including CMR in patients with COVID-19-related cardiac disease (Fig. 1), while slowly increasing the number of CMR studies performed in non-COVID patients with semi-urgent and elective indications.

### Purpose

**Fig. 2:**
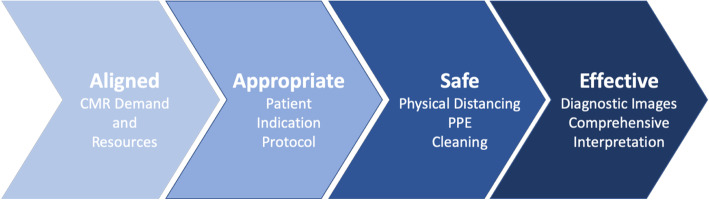
Key considerations for successful CMR COVID-19 re-activation. The CMR demand from referring services and the availability of resources (scanners,personal protective equipment (PPE), staff) must be aligned. A multidisciplinary, risk-benefit approach is required to select patients, indications, and protocols appropriately. The entire imaging pipeline including check-in, waiting, changing, and imaging must be safe and adhere to guidelines related to screening, physical distancing, masks, and cleaning. Finally, in order to be effective, CMR must result in diagnostic images with comprehensive interpretation

In this context, the overarching goal of this document is to provide a COVID-19 re-activation strategy specifically for CMR practices with the goal of offering CMR services in an environment which is as safe as possible for patients, technologists, physicians, and other frontline staff (Fig. 2).

### When is CMR re-activation appropriate?

Many national and local governments continue to provide guidance and regulations related to physical distancing protocols, stay-at-home and shelter-in-place orders, PPE precautions, and other regulatory matters. Moreover, local healthcare systems and hospitals are providing updated guidelines for patients, staff, and physicians who are transitioning back to more normalized healthcare delivery. The guidelines for CMR practice re-activation provided in this document are intended only as a supplement to these local governmental and health system guidelines which should be adhered to at all times.

When evaluating the appropriate timing to scale up semi-urgent and elective procedures, CMR units should consider coordinating with local and state public health and hospital system officials to review the availability of PPE for staff and patients, testing capacity, CMR facility readiness including physical distancing preparedness in reception areas, and workforce availability (including technologists, nurses, and interpreting physicians). Moreover, the needs of specific clinical services which are also re-activating should also be considered including other MRI specialties, electrophysiology, other imaging, interventional cardiology, heart failure, cardiac surgery, thoracic surgery, and vascular surgery [11]. Re-activation should proceed at a rate that appropriately balances the availability of resources with the clinical demand for CMR examinations and patient readiness.

### Determining appropriate CMR indications during re-activation

In the recent initial COVID-19 guideline document, the SCMR classified cardiovascular imaging examinations as urgent, semi-urgent, and elective, and advised that most non-urgent exams be delayed [9]. As the first peak phase passes, semi-urgent and elective CMR examinations can begin to be scheduled. The American College of Radiology (ACR) has provided guidance on the safe resumption of non-urgent diagnostic imaging, while a consensus document from multiple North American cardiovascular societies has outlined procedures for safe re-introduction of cardiovascular services [11, 12]. According to the ESCR, cardiac imaging programs should be firstly reopened to patients with “subacute” indications that were rescheduled during the pandemic, whereas pre-operative scans may be scheduled close to the surgical procedures [7]. In general, identifying appropriate examinations and scheduling time frames should be based on shared decision making between referring providers, CMR unit physicians and staff, and patients. While this approach is always recommended, the COVID-19 specific risks associated with imaging patients, including risks to frontline healthcare workers, must be weighed against the benefit of the examination. Thus, if a patient’s risk of illness or death from delaying CMR is greater than the risk of nosocomial transmission to healthcare workers and patients, then the CMR examination should be performed in a timely fashion.

A tiered plan for re-activation based on study indication and urgency will be useful for prioritizing patients for scheduling purposes. However, in order to limit potential exposures, other considerations should also influence scheduling of CMR examinations including additional patient appointments at the same facility, the potential impact of CMR findings on timing and availability of surgical procedures, and the availability of the CMR unit team including technologists and nursing. Patients whose CMR procedures were delayed as a result of the pandemic should also be prioritized as much as possible, but it is reasonable to re-assess the appropriateness of the exam and consider the availability or recent performance of alternative diagnostic modalities given the potentially long interval since the original imaging order was placed.

Once a particular patient and indication is deemed appropriate, the optimal CMR protocol must be carefully considered to ensure successful diagnosis and minimize the likelihood of patient callbacks or other delays in interpretation. Generally, CMR studies should be tailored to answer a particular clinical question(s) with the goal of creating time-efficient protocols [13]. However, in the context of COVID19, reducing redundancy in diagnostic testing can help limit exposure, and opportunities to expand CMR protocols should be considered in a multidisciplinary fashion. A representative example is the resumption of electrophysiology ablation procedures which require pulmonary vein mapping. If CMR pulmonary vein mapping is performed, the CMR protocol may be optimized to evaluate for left atrial appendage (LAA) thrombus by including long inversion time late gadolinium enhanced (LGE) images through the LAA, or targeted, post-gadolinium balanced steady state free precession (bSSFP) cine imaging [14, 15]. These modifications could reduce the need for dedicated transesophageal echocardiography for LAA thrombus evaluation and be particularly useful at high volume institutions. Other similar protocol modifications can be considered and may include the addition of stress perfusion CMR, dedicated valvular imaging, or CMR angiography as may be appropriate in a given patient with the goal of decreasing potential overall patient and healthcare worker COVID-19 exposure.

Additional strategies to consider include the implementation of rapid imaging protocols to reduce imaging times and improve throughput. Both the Impact of Non-invasive CMR Assessment (INCA) Peru study and the Rapid Cardiovascular Magnetic Resonance for Ischemic Heart Disease Investigation (RAPID-IHD) study have demonstrated the feasibility of acquiring localizer images, short and long axis segmented bSSFP cine images, and LGE with scan times averaging less than 20 min [16, 17]. Additional sequences such as native T1 or T2 mapping could then be easily added to this protocol in appropriately selected patients.

### Patient and healthcare worker COVID-19 safety precautions

#### Patient screening and COVID-19 status (Fig. 3)

Safety precautions which have been implemented during the peak-phase of the pandemic must continue including screening patients, healthcare workers, and, if allowed, visitors or patient support guests for COVID-19 symptoms and exposure. Most health systems have devised criteria based on current or recent symptoms and potential exposure history to help triage patients for further laboratory testing. Ideally, all patients, and particularly any patient with concerning symptoms or exposure history, as well as exposed healthcare workers should be evaluated with a real-time polymerase chain reaction (RT PCR) COVID-19 assay. However, local testing criteria may change over time based on the availability of tests and presumed local prevalence of disease. Pre-imaging screening for symptoms of COVID-19 infection or risk should be performed in all patients prior to CMR, including patients with a history of negative COVID-19 RT-PCR. This screening should ideally be performed by phone 1–2 days prior to the imaging appointment and confirmed at the time of patient arrival.

**Fig. 3:**
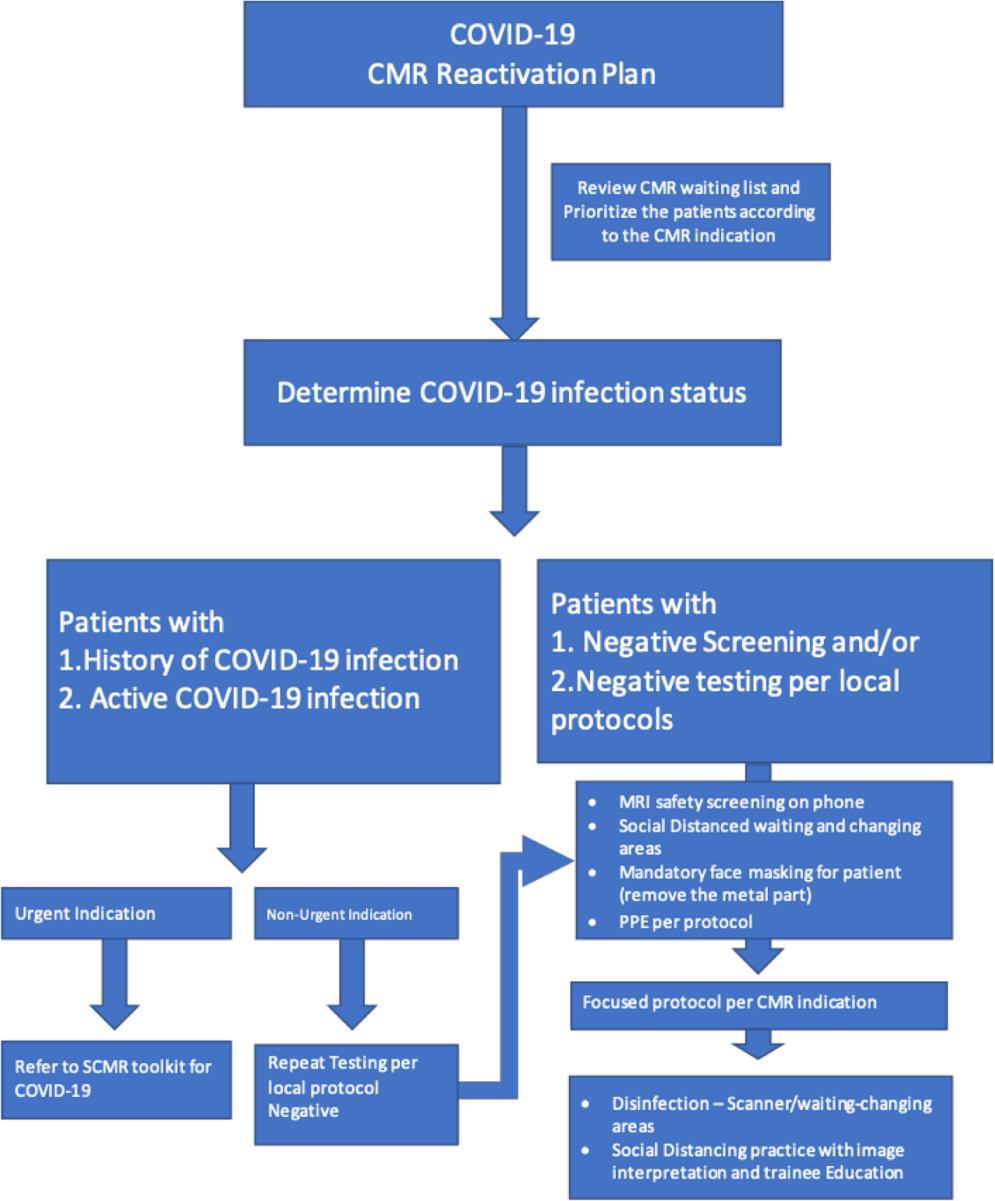
Overview of CMR reactivation COVID-19 screening protocol

Based on patient history, laboratory testing, and pre-imaging screening, three subcategories of patients can be defined: COVID-19 negative, COVID-19 positive (or presumed positive/patient under investigation), and recovered COVID-19.

COVID-19 negative patients include those that have no concerning symptoms or exposure history and may or may not have tested negative for COVID-19. These patients will generally present the lowest risk of transmission to frontline healthcare workers and other patients. In general, with appropriate screening and PPE precautions, these patients can resume CMR.

COVID-19 positive patients will generally be at higher risk of transmitting the disease to healthcare workers and other patients. This category should also include patients with COVID-19 symptoms or an exposure history within the last 14 days, as well as any patients currently being evaluated for COVID-19. This subgroup requires careful consideration of the risks and benefits of the CMR exam prior to performing images and, if possible, exams should be delayed until the patient has recovered. If delaying imaging would result in unacceptable delays in patient care, imaging should be scheduled at a time which will limit exposure to other patients and healthcare workers. Strategies to reduce interactions, provide adequate personal protective equipment, and sanitize the imaging facility should be implemented as outlined below.

The recovered COVID-19 patient subgroup is the most challenging to define and may reflect an increasing number of patients evaluated with CMR as the cardiac complications associated with COVID-19 infection continue to manifest. Several strategies for defining recovery have been suggested, including symptoms, time, or test-based strategies. Recommendations from the United States Centers for Disease Control and Prevention (CDC) and World Health Organization (WHO) define recovery as 1) 3 days since fever and respiratory symptoms have resolved, at least 10 days since first symptoms appears, and if available, two consecutive negative RT-PCR assays collected at least 24 h apart [18, 19]. If a patient never had symptoms but had a positive RT-PCR assay, at least 10 days are required between the first positive test and consecutive negative tests. Local health systems may develop their own strategies for defining recovery, and it currently is not clear how COVID-19 antibody testing may be used to better characterize this group. The understanding of infectivity, viral load, and immunity of recovered patients is evolving, although the risk of spreading the virus in patients without active symptoms is believed to be low [20, 21]. It is likely appropriate to proceed with CMR for recovered patients in most circumstances. However, it remains necessary to adhere to all recommended physical distancing and PPE guidelines, and to confirm that the patient has met recovery criteria and is currently asymptomatic prior to arrival at the CMR center.

#### Physical distancing

In accordance with guidelines from the CDC [22], physical distancing is necessary to reduce the spread of the virus and all waiting, check-in, and changing areas should be arranged to allow appropriate space between patients. In COVID-19 positive patients, isolated waiting and changing areas are necessary. For all patients, alternative waiting strategies could also be considered including waiting in cars while electronically checking in rather than in-person at facility waiting areas. COVID-19 screening and CMR safety screening should be performed over the phone *prior to patient arrival* to further reduce on-site time and unnecessary staff exposure to symptomatic patients.

Imaging schedules should be arranged to minimize patient overlap in waiting areas (e.g. staggered procedure start times), and to allow ample time for equipment cleaning between exams. These precautions will likely result in increased scan interval times and reductions in overall patient volume. Facility specific solutions including longer imaging hours could be considered as required by CMR demand, staff availability, and patient acceptance.

As much as is feasible, reducing unnecessary contact between individuals (patient-to-patient and patient-to-healthcare worker) during the imaging appointment should also be pursued. This may include additional workflow changes such as one-way walkways, and enhanced communication between imaging staff, transport teams, and others.

#### Personal protective equipment

Local guidelines for PPE should be followed including the gowns, gloves, masks, and face shields. Availability of appropriate PPE for frontline staff should be confirmed prior to increasing CMR volumes and the local stock of PPE should be regularly monitored.

Surgical masks for patients and healthcare workers should be utilized as much as possible [22]. Many health systems have mask requirements in place, but local guidance on mask type and who must be masked should be followed. The time-varying radiofrequency pulses and magnetic field gradients used in CMR are hazardous for patients with masks which contain small staples or metallic components [23] and the metal component should be removed prior to entry into the scan room. The ACR has specific recommendations related to the use of masks in MRI zone IV for patients and healthcare personnel [24]. In general, patients should be fit with MR-safe masks when available. If not available, standard surgical masks could have metal components removed, for example the nose bridge component, and the mask can then be secured with tape. If patients bring non-standard masks, part of the CMR safety check should involve assessing mask components and transitioning to CMR center provided masks. Patients should also be instructed to alert the technologist to burning or pain associated with the mask during the exam which may indicate heating or displacement of ferromagnetic components.

Since technologists and other healthcare workers will not be in zone IV during scanning, the main mask-related risk is magnetic forces on ferromagnetic components of the mask when entering or leaving the scanner room. Powered air-purifying respirators (PAPR) should not be brought into the scanner room as they may contain ferromagnetic components which can be damaged by the magnetic field.

#### Cleaning and sanitization

Scanners, waiting areas, and changing areas should all be cleaned in accordance with local agency guidelines and hospital system requirements [22]. Realistic cleaning times should be confirmed prior to re-activation to avoid unnecessary waiting and increased unnecessary patient-patient and patient-healthcare work contact.

#### CMR in COVID-19 patients or persons under investigation

Cardiovascular complications of COVID-19 are increasingly reported and there will likely be an increasing role for CMR to better characterize these findings. Please refer to the initial SCMR COVID-19 guidelines and online Preparedness Toolkit for a complete description of CMR imaging of COVID-19 patients or patients under investigation for COVID-19, including ventilated patients [9, 25].

### CMR image interpretation

Remote CMR image interpretation should provide the highest level of protection to CMR interpreting physicians. When this is not feasible, limiting the number of staff on site is recommended. When on site, physical distancing measures are necessary including isolated reading rooms and phone or text interactions with technologists, nurses, and other physicians is preferred.

While urgent studies may require some compromise in ideal image interpretation protocols, semi-urgent and elective imaging should only be pursued when a comprehensive interpretation is feasible. The interpreting physician should have access to necessary high-resolution screen, post-processing, 3D visualization, and cardiac functional analysis and tissue characterization software.

In patients with findings which are suspicious for COVID-19 infection including typical lung parenchymal findings [26], myocarditis [27], or pulmonary embolism [26], the ordering physician should immediately be made aware. Notification is especially important in patients who have not been diagnosed as COVID-19 positive and are likely at increased risk of spreading the disease. For cardiologist readers, suspicious extra-cardiac findings should be discussed with a cardiothoracic radiologist.

#### Trainee education

For CMR units in academic settings, re-integrating trainees into the scanner and reading room may be appropriate during re-activation. This process should, however, adhere to local trainee-specific COVID-19 guidelines and should never compromise trainee safety. Similar to interpreting physicians, trainees should work remotely as much as feasible with teaching performed remotely (e.g. phone or secure video conferencing). When on site, trainees should adhere to all physical distancing and PPE guidelines. The number of trainees working in a given unit should be proportional to the volume of work such that the education experience is valuable relative to the potential risks related to infection.

### CMR research

The resumption of CMR research should likely be performed using a phased approach with the overall goal for the safe and effective return to in-person research activities while adhering to local and institutional guidelines. The strategies in many cases should mirror those described above for clinical re-activation including comprehensive screening of research participants and staff, appropriate physical distancing practices and PPE utilization. Limiting the number of research staff in a specific area, such as a CMR reading room, control rooms, research offices, or laboratories at any given time is also recommended. In general, research activities should continue to be performed remotely as much as possible with in-person activities performed only as needed, making sure that equipment-cleaning measures have been defined, and the number of individuals involved in a given research visit minimized. Potential phases for return to research activities may include the initial resumption of phantom testing activities or pre-clinical animal research while adequate screening, physical distancing, and PPE protocols are being developed. Resuming human subjects research should likely focus initially on time-sensitive research studies such as clinical trials with pre-specified protocols, followed by less restrictive human subject research. Obviously, the risk of COVID-19 infection to subjects and staff should be considered when recruiting research subjects, and ideally subjects would undergo COVID-19 testing prior to imaging whenever possible. Subjects should be made aware of the safety protocols utilized by the research facility. Also, research team members should receive specific training on COVID-19 safety procedures.

## Conclusion

As the SARS-CoV-2 pandemic transitions out of the peak phase in most countries, it is appropriate for CMR units to begin performing many semi-urgent and elective CMR studies. Adherence to updated local institutional guidelines, while balancing the benefits of imaging with COVID-19 related risks to patients and healthcare workers should guide re-activation timing and patient selection.

## Data Availability

N/A.

## Abbreviations

ACR: American College of Radiology
bSSFP: Balanced steady state free precession
CDC: United States Centers for Disease Control and Prevention
CMR: Cardiovascular magnetic resonance
CMS: Centers for Medicare and Medicaid Services
ESCR: European Society for Cardiac Radiology
ESR: European Society of Radiology
INCA: Impact of Non-invasive CMR Assessment
LAA: Left atrial appendage
LGE: Late gadolinium enhanced
PAPR: Powered air-purifying respirators
PPE: Personal protective equipment
RAPID-IHD: Rapid Cardiovascular Magnetic Resonance for Ischemic Heart Disease Investigation
RT-PCR: Real-time polymerase chain reaction
SARS-CoV-2: Severe acute respiratory syndrome coronavirus 2
SCMR: Society for Cardiovascular Magnetic Resonance
WHO: World Health Organization
